# The Role of Binding Site on the Mechanical Unfolding Mechanism of Ubiquitin

**DOI:** 10.1038/srep08757

**Published:** 2015-03-04

**Authors:** Penghui Cao, Gwonchan Yoon, Weiwei Tao, Kilho Eom, Harold S. Park

**Affiliations:** 1 Department of Mechanical Engineering, Boston University, Boston, MA 02215; 2 Department of Mechanical Engineering, Korea University, Seoul 136-701, South Korea; 3 Biomechanics Laboratory, College of Sport Science, Sungkyunkwan University, Suwon 440-746, South Korea

## Abstract

We apply novel atomistic simulations based on potential energy surface exploration to investigate the constant force-induced unfolding of ubiquitin. At the experimentally-studied force clamping level of 100 pN, we find a new unfolding mechanism starting with the detachment between *β*_5_ and *β*_3_ involving the binding site of ubiquitin, the Ile44 residue. This new unfolding pathway leads to the discovery of new intermediate configurations, which correspond to the end-to-end extensions previously seen experimentally. More importantly, it demonstrates the novel finding that the binding site of ubiquitin can be responsible not only for its biological functions, but also its unfolding dynamics. We also report in contrast to previous single molecule constant force experiments that when the clamping force becomes smaller than about 300 pN, the number of intermediate configurations increases dramatically, where almost all unfolding events at 100 pN involve an intermediate configuration. By directly calculating the life times of the intermediate configurations from the height of the barriers that were crossed on the potential energy surface, we demonstrate that these intermediate states were likely not observed experimentally due to their lifetimes typically being about two orders of magnitude smaller than the experimental temporal resolution.

Mechanical force has recently been utilized to mimic the effects of external stimuli that cause the folding, unfolding and misfolding of biological proteins^1^,^2^. This has become possible over the past 15 years due to the recent experimental advances based on force clamping techniques using atomic force microscope (AFM) and optical tweezers (OT), which can apply a constant force to each end of a protein in order to obtain the force-dependent unfolding time distributions^1^,^3^,^4^,^5^,^6^. These experiments have been used not only to force the proteins to explore new, previously unaccessible portions of the potential energy landscape, but also to determine if the unfolding configurations can be correlated to those seen due to chemical denaturation^7^,^8^.

One protein that has been extensively studied using such single molecule extensional experiments is ubiquitin, which consists of 76 amino acids, and is known to regulate diverse inner-cellular processes^9^. Ubiquitin's sole functional binding site exists at Ile44, which can interact non-covalently with the ubiquitin binding domains that exist in enzymes for activating, conjugating and ligating^10^. The force clamping experiments have reported that the time required to completely unfold ubiquitin is a few seconds when the applied force is 100 pN^11^,^12^. Furthermore, by performing an extensive number of such experiments, Fernandez et al. reported that ubiquitin exhibits intermediate states when the end-to-end extensions are about 8 and 12 nm^12^.

However, a key shortcoming of single molecule pulling experiments is the lack of atomistic detail with regards to the unfolding pathways, as well as the structure of the intermediate configurations^13^. Researchers have thus used steered molecular dynamics (SMD) simulations to obtain such details^14^, with the key shortcomings that SMD simulations occur at forces that are significantly larger and at timescales that are significantly shorter than occur experimentally^15^,^16^,^17^. Other researchers have used monte carlo (MC) simulations^18^ with simplified force fields, or coarse-grained models^19^,^20^,^21^,^22^ to study the unfolding at experimentally-relevant force ranges. While these simulations have found intermediate configurations with end-to-end extensions matching those reported experimentally, they also suffer from the drawbacks that neither the Go-like models nor simplified potentials are as accurate as all-atom simulations, and also that Go-like coarse-grained models are unable to capture the sequence-specific protein unfolding mechanisms as are described in the present work.

Due to these known issues with SMD, other researchers have studied the protein folding and unfolding problem from the viewpoint of potential energy surface (PES) exploration. This viewpoint does not require *a priori* information about the possible transitions or intermediate states a protein can take, but instead finds them by searching out the different energetic transitions that are seen in its multi-dimensional PES. One example of such an approach is the discrete path sampling (DPS) method of Wales^23^,^24^, who used PES exploration to determine rate constants for different transition states. Connections between the energy barriers crossed on the PES and time (or rate) were then made using transition state theory, enabling the extension of these atomistic methods to experimental time scales. This approach was later utilized in conjunction with static applied forces to demonstrate how the PES changes as a function of the direction of the applied force^25^ for proteins L and G, and also to study the folding and unfolding of proteins and peptides at experimental time scales^26^,^27^,^28^.

In this work, we employ the self-learning metabasin escape (SLME) method^29^,^30^,^31^, which couples potential energy surface exploration with constant applied forces to study the mechanical unfolding of ubiquitin at experimentally-relevant forces (around 100 pN), and time scales (seconds). The SLME method is similar in respects to the DPS approach of Wales in that exploration of the PES is utilized to find energetic barriers between important energy minima, with the mapping from energy to time made using transition state theory, and also in the assumption that the dynamics must be Markovian, i.e. that transitions on the PES are independent from previous jumps. It is different in the approach used to explore the PES; the SLME method uses a basin filling approach, in which quartic penalty functions are used to flood an energy well, thereby forcing the system to exit and explore other neighboring wells, while the DPS methods use eigenvector-following techniques to seek transition states. The SLME method is also different in that its development has focused on application to problems involving mechanical deformation. Thus, connections between energetic barriers on the PES and mechanical strain rate have previously been established^31^.

In doing so, we report the novel finding that the binding site of ubiquitin, the Ile44 residue, is responsible for not only ubiquitin's biological functions such as ligand-binding, but also for its unfolding dynamics. Moreover, we report novel intermediate configurations that correspond to the end-to-end extensions observed experimentally as well as their related timescales for the key unfolding processes, which explains why the intermediate configurations of ubiquitin have generally not been observed experimentally.

## Results

All results in this section were obtained using the SLME method. The details of the method are given in the Methods section, while discussion about the method as well as comparison and validation against SMD simulations are given in the Methods section, as well as the Supplementary information. Specifically, we have verified that the SLME method gives the same unfolding pathways as SMD simulations at high (i.e. >300 pN) clamping forces, that the clamping force-dependent unfolding times are quite similar, and that the potentials of mean force around the unfolded configurations have the same force-dependence as was recently observed in low-force SMD simulations^32^.

**Figure 1:**
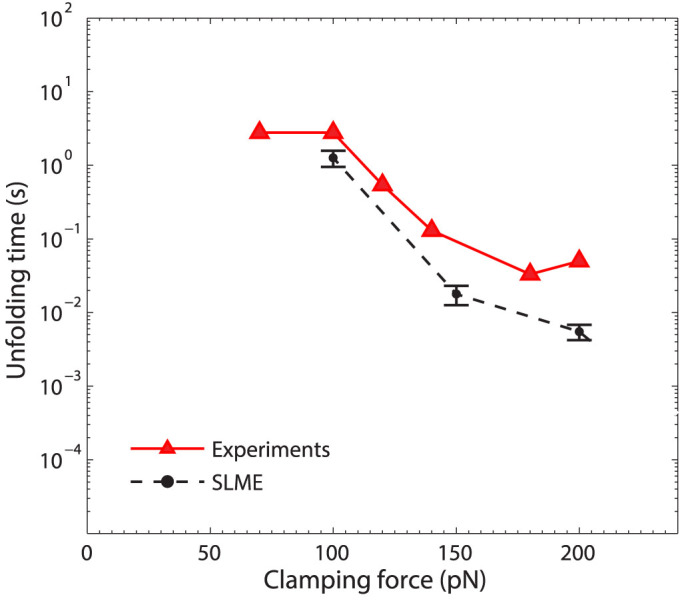
The unfolding time as a function of the clamping force obtained by the SLME method (black circle) in the low force regime. Each unfolding time is obtained by an average of at least 10 simulations for each clamping force. The experimental data (red triangles) is from Schlierf et al.^12^.

We first show in Fig. 1 that the unfolding times obtained using the SLME method are comparable to the experimentally obtained values in low force regime (*τ* = 10^−3^ to 10 s). Specifically, using the SLME method we find unfolding times on the order of seconds when the clamping force reduces to about 100 pN, which is similar to those reported in previous experimental studies^12^.

**Figure 2:**
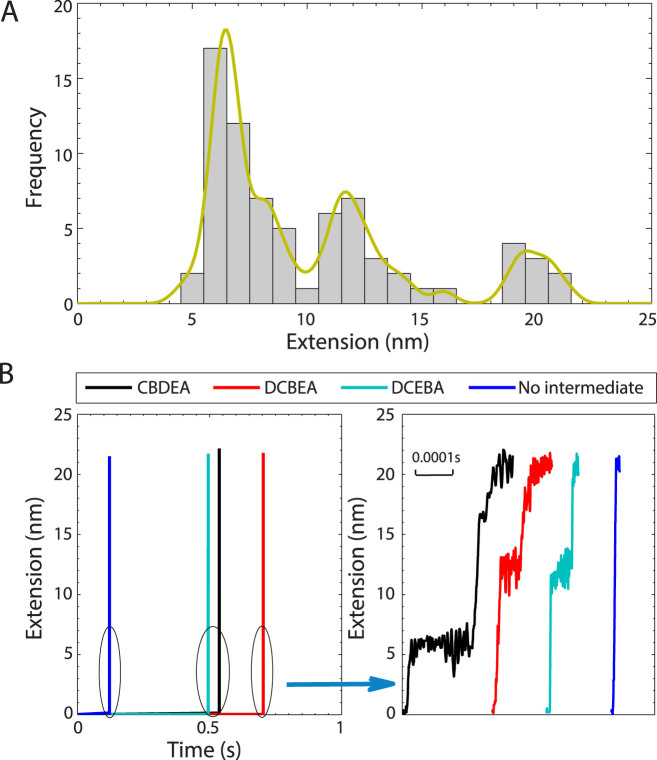
The mechanical unfolding of ubiquitin at 100 pN as obtained using the SLME method. (A) Frequency histogram of step sizes. (B) End-to-end extension as a function of time for four most common unfolding pathways. Right hand figure shows zoom in detailing the lifetime of the intermediate configuration.

Having established the ability of the SLME method to match both the MD and experimentally-observed unfolding times across a wide range of clamping forces, we focus our discussion on unfolding at 100 pN, as extensive experimental^12^ and computational^18^ studies were performed for this force value. To get a statistical representation of the unfolding pathways, we conducted 80 SLME simulations with the clamping force set as 100 pN, with the results reported in Fig. 2(A). First, unlike in the experiments^12^ and MC simulations^18^, we observe that at 100 pN the vast majority of the unfolding pathways pass through an intermediate configuration, as shown in Fig. 2(A). This is representative of a more general trend, shown in Fig. S4(B), where the percentage of unfolding simulations that pass through an intermediate state increases dramatically once the clamping force decreases below about 300 pN. This result is in contrast to the experimental results of Schlierf et al.^12^ and the MC simulations of Irback et al.^18^. We will explain and justify the results in Fig. S4(B) once we have introduced the new intermediate configurations we have uncovered below.

We now proceed to characterize the intermediate configurations that we observe using the SLME method, which are summarized in Fig. 2(B) for the 100 pN clamping force that was previously used experimentally^12^. Specifically, Fig. 2(B) shows the four most common unfolding pathways we observed, where paths CBDEA, DCBEA and DCEBA all involve an intermediate configuration, and thus three-state unfolding, while paths CBDEA and DCBEA also exhibit two state unfolding with no intermediate configuration. The alphabetic nomenclature we use to describe the different *β* sheets of ubiquitin follows that done previously^18^, and can be seen in the Supplementary Material, Figure S1.

Furthermore, we observe, similar to Schlierf et al.^12^, intermediate configurations centered around about end-to-end extensions of 7 and 12 nm, which correspond to representative paths CBDEA and DCBEA, respectively, in Fig. 2(B). The pathway DCEBA does not exhibit a consistent intermediate length, instead showing a distribution of intermediate extensions from 7 to 12 nm as shown in the supplemental information, Fig. S6. In addition, Fig. 2(B) also shows the unfolding time for each pathway, as well as the the lifetime of the intermediate configuration for one unfolding simulation. Because 80 different SLME simulations were performed, a distribution of the intermediate state lifetimes was obtained. Discussion about this and the connection to the energy barriers crossed on the potential energy pathway will be discussed later.

The occurrence of each pathway as a function of clamping force is summarized in Table 1. At high (>300 pN) clamping forces, the two-state CBDEA unfolding pathway is nearly always followed. As the clamping force decreases below 300 pN, the CBDEA three state unfolding pathway is observed more frequently, as are the two new unfolding pathways DCBEA and DCEBA. Overall, at 100 pN, two state unfolding is observed in just 11.3% of the simulations, with three state unfolding observed in 88.7% of the simulations. Interestingly, the novel unfolding pathways DCEBA and DCBEA are observed with equal probability (total 41.5%) as the lower energy three state CBDEA pathway (41.3%).

**Figure 3:**
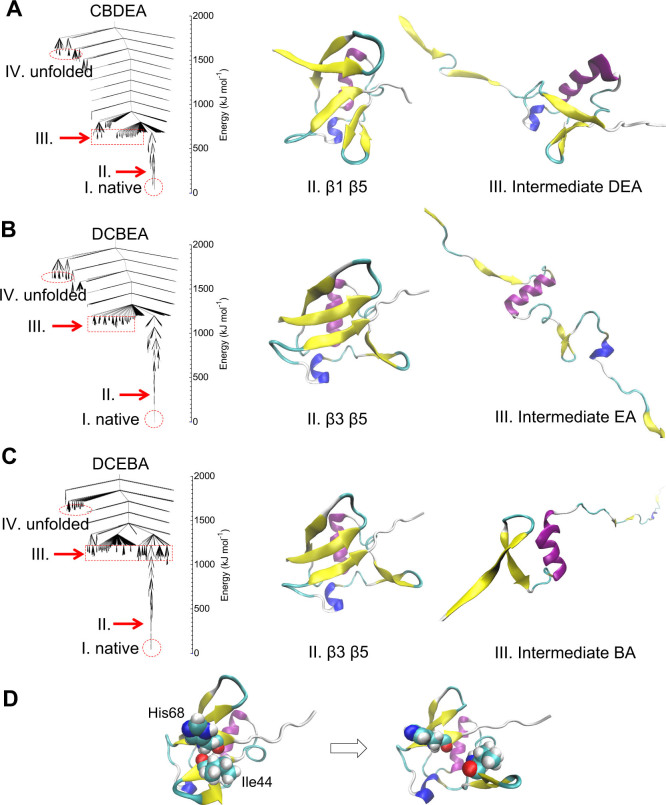
Disconnectivity graphs (left) of ubiquitin unfolding at 100 pN and the molecular structures (middle, right) for the first unfolding event and the resulting intermediate configuration. (A) CBDEA unfolding. II shows the separation of *β*_1_*β*_5_, while III shows the intermediate configuration. (B) DCBEA unfolding. II shows *β*_3_*β*_5_ detachment acts as the first unfolding event, with the resulting intermediate configuration shown in III. (C) DCEBA unfolding. II again shows *β*_3_*β*_5_ detachment as the initial unfolding event, while III shows the resulting intermediate configuration. (D) Demonstration of initiation of D unfolding pathway involving rotation of *β*_3_, including the Ile44 residue.

Fig. 3 shows the potential energy disconnectivity graphs for the unfolding pathway at 100 pN as well as corresponding atomic structures corresponding to the first unfolding event and the resulting intermediate configuration. We note that while we show the potential energy disconnectivity graphs here, approaches have been developed to show the free energy disconnecitivity graphs, for example as shown by Krivov and Karplus^33^, and also Evans and Wales^34^. In Fig. 3(A), the unfolding pathway is CBDEA with DEA as the intermediate configuration. Both the initial unfolding event of *β*_1_*β*_5_ separation as well as the intermediate state (DEA), which is found after detachment of *β*_1_*β*_2_, are comparable to earlier MC results^18^.

However, the two pathways that start from the unfolding of D, which corresponds to the detachment of *β*_3_*β*_5_ in Figs. 3(B) and (C), represent novel unfolding pathways at 100 pN as compared to previously proposed pathways^12^,^18^,^19^. Specifically, these unfolding pathways do not start with the *β*_1_*β*_5_ detachment that has been reported in all previous simulations of ubiquitin unfolding^12^,^18^,^19^. As shown in Fig. 3(D) for both the DCBEA and DCEBA pathways, the key event is the rotation of *β*_3_, which contains the Ile44 residue. The rotation of *β*_3_ first causes the separation of His68 on *β*_5_ with Ile44 on *β*_3_, and continues until *β*_3_ bonds with the distal part of *β*_5_. This sequence of events results in the initial unfolding event at 100 pN force starting from D (*β*_3_*β*_5_) rather than the previously reported unfolding pathway starting from C (*β*_1_*β*_5_), which was observed by us at higher clamping forces and by others at 100 pN^18^,^19^,^35^.

Furthermore, after the unfolding of *β*_3_*β*_5_, the breaking mechanism of *β*_1_*β*_5_ is also different from the unfolding in the high force regime. Specifically, at low forces, the separation of *β*_1_ and *β*_5_ occurs via tearing of the hydrogen bonds, while at high forces, the separation of *β*_1_ and *β*_5_ via shearing of the hydrogen bonds. Moreover, the potential energy for this new unfolding pathway, which starts from D in the low force regime as shown in Figs. 3(B) and (C), is clearly higher than that required in the CBDEA unfolding pathway with intermediate end-to-end extension of 7 nm in Fig. 3(A), as additional thermal energy is required for the rotation-induced detachment of *β*_3_*β*_4_ in Fig. 3(D). This thermal energy makes a greater contribution at the lower forces, which is why this mechanism is not observed at the higher forces that are applied in the SMD simulations.

It is also interesting to note some similarities between our new D-based unfolding pathways and recently reported folding pathways for ubiquitin. Specifically, in a recent all-atom MD simulation of high-temperature folding of ubiquitin, Piana et al. reported the existence of partially formed *β*_3_ and *β*_4_ with low Φ-values at the transition state ensemble^36^. Their MD simulations were run for a few milliseconds, which is important as the time scales we find for the unfolding events and intermediate configurations at 100 pN are on the order of tens or hundreds of microseconds.

For the D unfolding pathways, we observe that the detachment between *β*_3_ and *β*_5_ starts from the hydrogen bonds between Ile44 and His68. To determine the role of the Ile44 residue in the D unfolding pathway, we consider the mutation of Ile44 to Glycine, denoted as I44G. We performed 15 independent SLME simulations at 100 pN clamping force using the I44G mutation, and found that unfolding did not start from D (*β*_3_*β*_5_), but instead from C (*β*_1_*β*_5_) for all 15 cases.

To understand why the mutation prevents the D unfolding pathway, we compare the molecular structure near *β*_3_ and *β*_5_ for I44G and wild type ubiquitin. Specifically, we find the formation of a new hydrogen bond between Thr66 in *β*_5_ and Ala46 in the loop connecting *β*_3_ and *β*_4_ in I44G. The new hydrogen bond strengthens the *β*_3_*β*_5_ connection and also restricts the separation of Gly44 and His68, which thus prevents the initiating mechanism for the D unfolding pathway. Furthermore, the Gly residue has a smaller side chain than the Ile residue, which gives more flexibility to the molecular structure surrounding Gly, which enables the formation of a new hydrogen bond between *β*_5_ and the loop. Conversely, the large side chain of Ile gives less flexibility to structure surrounding Ile, which prevents the hydrogen bond formation between *β*_5_ and the loop in wild type ubiquitin. Overall, this mutation study has demonstrated the importance of the Ile44 side chain in enabling the new D unfolding pathway we have described.

The involvement of the Ile44 residue is important not only because it is the sole binding site of ubiquitin^9^,^10^, but also because it has been reported that protein binding sites exhibit relatively high flexibility with respect to the termini of a protein^37^, where predictions of this relatively high fluctuation of specific drug binding sites in proteins using the Gaussian network approach^38^,^39^ have been made. Previously, the binding sites of several proteins have been observed to adapt and reconfigure themselves depending on the binding molecule according to the induced fit model of conformation changes^40^. However, we observe conformational changes of the Ile44 binding site in an entirely different context in the present work, as the initial event enabling the new unfolding pathway, which is observed in the low clamping force regime of ubiquitin unfolding.

**Figure 4:**
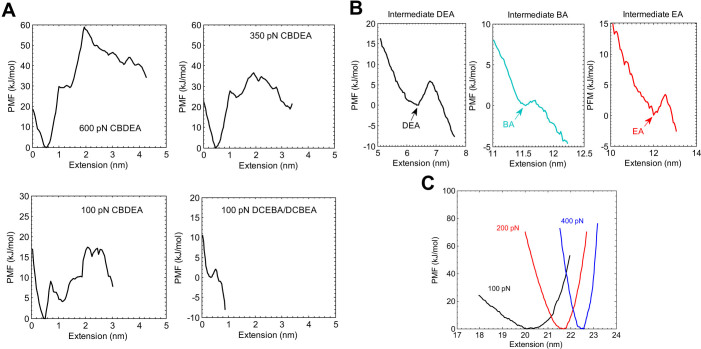
(A) PMF as a function of extension for the early stages of unfolding for different pathways, where 0 extension denotes the native configuration. (B) PMF for intermediate states shown in Fig. 1. (C) PMF at different forces for the fully unfolded states.

We show in Fig. 4 the potentials of mean force (PMF) that were calculated using umbrella sampling (see Methods for details). The umbrella sampling was done every 0.1 nm at a force constant of 1000 kJ mol^−1^ nm^−2^. Fig. 4(A) focuses on the initial *β* sheet separation, where the CBDEA pathways feature, at an extension of about 2 nm, breaking of *β*_1_*β*_5_ for the 600, 350 and 100 pN clamping forces. Furthermore, the energy barrier to breaking *β*_1_*β*_5_ decreases with decreasing force from about 60 kJ/mol at 600 pN to about 10 kJ/mol at 100 pN, which is due to the longer unfolding time required for smaller clamping forces, where the longer unfolding times give the system a higher probability of climbing over large energy barriers on the potential energy surface.

It is seen that the energy barrier that must be overcome to break *β*_1_*β*_5_ for the CBDEA pathway is much larger than is observed for the 100 pN DCEBA/DCBEA pathway shown at the bottom right hand corner of Fig. 4(A), where the *β*_1_*β*_5_ separation for the DCEBA/DCBEA pathways occurs at an extension of about 0.6 nm. The energy barrier for the *β*_1_*β*_5_ separation for the DCEBA/DCBEA pathways is lower due to the separation of *β*_3_*β*_5_ before the *β*_1_*β*_5_ separation occurs at 0.6 nm extension. Essentially, the initial detachment of *β*_3_*β*_4_ reduces the structural integrity of *β*_5_, such that the *β*_1_*β*_5_ separation requires less energy.

Fig. 4(B) shows the PMFs for the three main intermediate configurations observed in our simulations. The existence of each is verified by locating an energy barrier to be overcome along the unfolding pathway after the initial *β*-sheet separation. The PMFs of the intermediate configurations are presented, where (DEA) has the largest free energy barrier, while BA has the smallest, which will be connected to the lifetimes of each intermediate configuration. Fig. 4(C) shows the PMFs for three different force levels indicating that the extension needed to fully unfold ubiquitin increases with increasing force, a result that is consistent with previous MD simulations^32^ and what was observed previously in Fig. S4(A).

**Figure 5:**
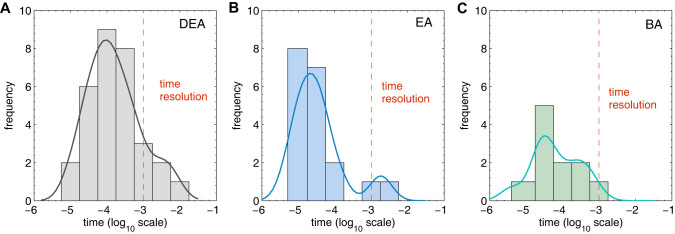
Frequency histogram of the survival times of the three most common intermediate configurations at a 100 pN clamping force. (A) CBDEA; (B) DCBEA; (C) DCEBA. Dashed red line corresponds to experimental temporal resolution limit of 1 ms^1^.

Finally, we discuss the seeming discrepancy between the large percentage of intermediate configurations (nearly 90%) observed in this work at 100 pN clamping force, which is in contrast to the nearly universal two state unfolding that was observed experimentally^12^. Specifically, we show in Fig. 5 the frequency histogram of the survival times of the three most common pathways (CBDEA, DCEBA, DCBEA) in which an intermediate configuration was found. These survival times were obtained by calculating the inverse of the rates of all small barriers crossed on the PES using transition state theory, as shown in Eq. 1 in the Method section. It is thus consistent with the previous discussion on the PMF barriers seen in Fig. 5(B) that, due to having the highest energy barrier to overcome to leave the intermediate state, the DEA intermediate has the longest lifetime as seen in Fig. 5(A).

As seen in Fig. 5, most of the survival times for all three intermediate configurations are smaller by about two orders of magnitude than the experimental resolution of 1 ms^1^, or −3 on the log_10_ scale used in Fig. 5, which may explain why only a small number of intermediate configurations were found in experimental studies of force clamp unfolding of ubiquitin^12^. This result is also consistent with recent SMD simulations of the force-induced unfolding of ubiquitin^32^, in which no intermediate configurations of ubiquitin were observed for clamping forces ranging from 30 to 250 pN, likely because the total simulation times were 150 nanoseconds or less, which is much smaller than the survival time for any of the intermediate configurations we have reported.

## Conclusions

In summary, we have used new atomistic simulation methods that can access experimentally-relevant force clamping values and time scales to study the mechanical unfolding of ubiquitin. Our novel finding is that our atomistic simulations have uncovered the role of amino acid sequence in protein dynamics and mechanics such as protein unfolding dynamics. Specifically, we have uncovered a new unfolding pathway whose initial event involves large conformational changes of the *β*-sheet containing Ile44, the sole binding site of ubiquitin. As indicated in this work, the functional site of ubiquitin (for instance, the binding site) is responsible for not only its biological function such as ligand-binding but can also impact its unfolding dynamics. When the Ile44 residue serving as the ubiquitin binding site is mutated, we found different unfolding pathways, which implies that a single point mutation may lead to undesirable folding process such as protein misfolding.

Due to the ability of our atomistic simulation to probe the long-time dynamics and mechanics of protein structure as described in this work, our work can be further extended for studying the force-driven protein dynamics such as protein unfolding mechanics as well as force-quenched protein refolding dynamics^11^, whose relevant time scales are much larger than those of classical MD simulations. Here, it is noted that this force-quenched refolding dynamics may allow one to quantitatively characterize a protein misfolding related to diseases such as amyloidosis^41^. Our work suggests that the SLME simulations enable not only long-time simulation of protein dynamics with time scales close to those accessible with single-molecule experiments, but also an insight into the role of amino acid sequence on the long-time protein dynamics such as protein (un)folding, refolding/misfolding, and protein aggregation.

## Methods: Self-Learning Metabasin Escape Algorithm

It is well-known that steered MD (SMD) simulations for force clamping exhibit two major drawbacks as compared to the corresponding force clamp experiments. First, the SMD simulations are typically performed at constant applied forces (i.e. greater than 300 pN) that are much larger than those used experimentally (100 pN) for ubiquitin^42^. While some recent MD simulations have used experimentally-relevant unfolding forces^32^, this does not resolve the issue that the time scales that are accessible with SMD are limited to the order of hundreds of nanoseconds, which is more than 7 orders of magnitude smaller than the experimentally-observed unfolding time of seconds^12^. Thus, the energy landscape explored during steered MD simulations is likely to be different than that in experiments^42^.

As a result of the known time scale shortcomings intrinsic to SMD simulations, there has recently been a burst of effort in developing other atomistic simulation techniques to circumvent the time scale problem. One such approach is the autonomous basin climbing (ABC) approach^29^, which is a metadynamics-like approach^43^ that has recently been used to probe extremely slow dynamical processes like nanocrystal creep^44^, void nucleation and growth^45^ and dislocation-defect interactions at slow strain rates^46^. The ABC method works by adding Gaussian-like penalty functions to the PES to force the system to climb out of energy wells and explore other, neighboring parts of the PES. However, the ABC method, as well as other PES activation techniques, suffers from an increase in computational expense as more of the PES is explored due to the need to history-penalize the PES, which means that penalty functions must be stored such that regions of the PES that have already been explored are not visited again. One manifestation of this is that while longer time scales can be achieved, the strain rates that are achieved are still higher than those in experiments^46^.

The self-learning metabasin escape (SLME) method represents an improvement to the ABC method in which penalty functions are applied to the PES, but where the penalty functions in a given energy well are combined such that, upon exiting the well, only a few penalty functions remain to prevent the system from re-entering the well^30^. This simple combination scheme was shown to significantly improve the computational efficiency. The computational efficiency gained enabled the study of both larger atomistic system sizes, as well as the ability to climb over the large energy barriers that are needed in order to access long, experimentally-relevant time scales via connection to transition state theory^30^,^31^,^47^.

For application of the SLME method to protein unfolding, quartic penalty functions are utilized to push the system out of potential energy wells in which it can become stuck due to the relatively low clamping force (100 pN) that is constantly applied. It is worth noting that the SLME results do depend on the choice of the free parameters of the method, namely the width and height of the penalty functions. These parameters were chosen by comparing with benchmark high force SMD results. In doing so, it was found that choosing initial widths in the range of 0.1 to 1.6 nm, and height of 20 kJ/mol led to results that both agreed well with the SMD results, but also enabled reasonable computational costs.

Application of the quartic penalty functions is followed by an energy minimization. Upon application of a sufficient number of penalty functions, the system escapes over the lowest energy barrier to a neighboring potential energy well, where penalty functions are again applied if the applied force is not sufficient to lower the energy barrier to enable the system to escape. By comparing the energy of all energy minimized configurations, the barriers *Q* separating two local energy minima can be obtained. Furthermore, while the SLME method typically exits a given energy well via the lowest energy barrier, in practice penalty functions are applied with non-constant widths and heights within a reasonable range to introduce a degree of stochasticity into the PES search, which enables us to find alternate unfolding pathways, as shown in the main manuscript. Because the penalty functions are only needed if the applied clamping force is not sufficient to lower the energy barrier by itself, the penalty functions can be physically interpreted as thermal activation that assists the mechanical force in enabling the system to escape from a local energy minimum. This procedure is repeated until ubiquitin is completely unfolded such that its end-to-end length is about 23 nm at 100 pN^32^. In going through this procedure, the system is able to find and pass through all relevant intermediate configurations, which are described in the main manuscript.

The unfolding time is estimated using transition state theory^47^,^48^ via
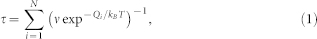
where *Q_i_* is the energy barrier separating energy minima *i* − 1 and *i*, *N* is total energy minima explored on the unfolding path, *ν* is a frequency prefactor and *T* is the temperature. We used the value *ν* = 4 × 10^9^ s^−1^ for the low force (<300 pN) regime that was recently obtained experimentally for the mechanical unfolding of ubiquitin^48^. We note that the choice of a constant prefactor for all of the barriers crossed on the PES is not the most accurate one, as other approaches exist that enable the calculation of the prefactor for each barrier directly from information that can be obtained during the PES exploration. Such work has been performed recently by Stevenson and Wales^49^ and Wales^50^. However, the choice of the constant, experimentally-determined prefactor in this work does result in qualitatively accurate estimates for the unfolding time over a wide range of forces as compared to the previous experimental data^12^.

We employed the AMBER99sb potential field^51^ which utilizes an implicit solvent model for water in conjunction with the Protein Data Bank (PDB) ID 1UBQ for the native configuration of ubiquitin. The native ubiquitin structure was equilibrated at 300 K, and then energy minimization was performed to generate the corresponding local energy minimum. At both the N and C-termini we applied a constant pulling force ranging from 100 to 600 pN using the SLME method. Clamping forces greater than 300 pN were also simulated using SMD to compare with the SLME results for higher forces. All simulations, i.e. both SMD and SLME, were performed using the open source GROMACS simulation package^52^.

For obtaining the PMF, we did umbrella samping with 100 pN clamping force using GROMACS. We sampled every 1 Å of end-to-end distance with 1000 kJ-mol^−1^Å^−2^ force constant during 1 ns equilibration. The PMF was then extracted by the Weighted Histogram Analysis Method (WHAM)^53^.

We performed extensive validation of our SLME method, with the focus on comparing the results obtained using the SLME method to those obtained using SMD at high constant forces ranging from 300 to 600 pN. The first validation is to compare the unfolding time. As shown in Fig. S3, the unfolding time as a function of large clamping forces ranging from 300–600 pN obtained from SLME simulations is comparable to that estimated from SMD simulations. Furthermore, the trend that the unfolding time increases for decreasing clamping force is also captured by both simulation techniques.

The second validation is to compare the unfolding mechanism. For the high force regime of 300–600 pN, we observe only two states, i.e. the initial folded (native) configuration and the unfolded configuration. Furthermore, in Fig. S2, the unfolding pathway for the high force regime is presented as CBDEA where *β*_1_*β*_5_ is C, *β*_1_*β*_2_ is B, *β*_3_*β*_5_ is D, *β*_3_*β*_4_ is E and *α* is A (see Fig. S1 for structural definition). This unfolding pathway is the same as previously reported using MC simulations^18^, and is found using both the SMD and SLME approaches.

Finally, we compare the force-dependent PMFs for the unfolded configuration, as shown in Fig. 4(C). There, we observe two characteristics that are similar to those recently reported for low clamping force SMD simulations^32^. First, the extension at the unfolded state increases with increasing clamping force. Second, the steepness of the energy well increases with increasing clamping force; both of these trends were also reported by Stirnemann et al.^32^. All of these characteristics indicate the ability of the SLME approach to capture the same unfolding phenomena in SMD simulations at large clamping forces.

## Author Contributions

P.C. and G.Y. made equal contributions to this work. H.S.P., K.E. and G.Y. designed the study. P.C. and W.W.T. performed the SMD simulations. P.C. developed the SLME algorithm and performed the simulation, H.S.P., G.Y., P.C., W.T. and K.E. analyzed the results and wrote the manuscript.

## Supplementary Material

Supplementary Information Supplemental Information

## Figures and Tables

**Table 1:** Relative frequency of unfolding pathways for different clamping forces. 80 simulations were performed at 100 pN, 30 for the other force values

Force (pN)	3 state unfolding			2 state unfolding	
	CBDEA	DCBEA	DCEBA	CBDEA	DCBEA
100	41.3%	27.5%	13.8%	5.0%	6.3%
150	33.3%	13.3%	-	23.3%	16.7%
300	13.3%	-	-	86.7%	-
600	-	-	-	100%	-
