# Supplementary material for: The Role of Binding Site on the Mechanical Unfolding Mechanism of Ubiquitin
Source: Sci Rep. 2015 Mar 4;5:8757. doi: 10.1038/srep08757 (PMC4348633; doi:10.1038/srep08757)
Supplement: Supplementary Information — Supplemental Information [file srep08757-s1.pdf]

Supplementary Material for The Role of Binding Site on the Mechanical Unfolding Mechanism of Ubiquitin

Penghui Cao,[1,*] Gwonchan Yoon,[1,2,*] Weiwei Tao,[1] Kilho Eom,[3] and Harold S. Park[1,†]

[1]*Department of Mechanical Engineering, Boston University, Boston, MA 02215*
[2]*Department of Mechanical Engineering, Korea University, Seoul 136-701, South Korea*
[3]*Biomechanics Laboratory, College of Sport Science, Sungkyunkwan University, Suwon 440-746, South Korea*

I. VALIDATION OF SELF-LEARNING METABASIN ESCAPE ALGORITHM VIA COMPARISON TO STEERED MD

Our first objective is to give further details on the accuracy of the self-learning metabasin escape (SLME) method [1, 2, 4] by comparing its results to steered molecular dynamics (SMD) simulations. Both SLME and SMD simulations were performed on ubiquitin, whose structure is illustrated in Fig. S1, for a force clamp ranging from 350 to 600 pN. In both the SMD and SLME simulations, we employed the AMBER99sb potential field with the GB/SA implicit solvent model. All SMD and SLME simulations were performed using the publicly available GROMACS simulation code.

Starting from the native configuration for ubiquitin, energy minimization was first performed to equilibrate the structure, followed by a 1 ns equilibration within the NVT ensemble at 300 K. After the minimization and temperature equilibration, the clamping force was applied at both the N and C termini of ubiquitin. The unfolding pathway obtained using SLME for a force clamp of 350 pN is shown in Fig. S2, which is the same as obtained using the SMD simulations. This is also observed for the other clamping forces we considered in comparing SMD and SLME, i.e. at 500 and 600 pN.

A comparison of the unfolding time for ubiquitin as computed using SMD and SLME approaches is given in Fig. S3 for clamping forces ranging from 350 to 600 pN. As can be seen, the unfolding time increases as the clamping force decreases, with the MD and SLME approaches in good agreement both in terms of the unfolding time, as well as the trend in the unfolding time. Furthermore, both MD and SLME predict that, for these clamping forces, all unfolding pathways follow two-state behavior.

Fig. S4 (A) shows the unfolding step size for representative SLME simulations at different clamping forces. The unfolding pathways at 400 pN clearly exhibit two-state characteristics. In contrast at 150 pN, some unfolding pathways pass through an intermediate state. The general trend is illustrated in Fig. S4 (B), where it is shown that the percentage of unfolding simulations that show an intermediate state increases dramatically as the clamping force decreases below about 300 pN.

Another point of comparison is demonstrated by Fig. 4(C) in the main manuscript. There, we calculated the PMFs for different applied clamping forces around the unfolded configuration. The results are very similar to those recently published by Stirnemann et al. [6]. First, the extension at the unfolded state increases with increasing clamping force. Second, the width of the PMF around the unfolded state decreases with increasing clamping force, again in agreement with the earlier findings [6].

II. 100 PICONEWTON FORCE CLAMP

We focus now on providing additional details for the 100 pN force clamp, which is the force level at which novel intermediate configurations were reported in the main manuscript. We first show in Fig. S5 a plot showing the end-to-end extension as a function of the energy minima that were explored along the unfolding pathway, for the four unfolding pathways we observed for this clamping force.

We show in Fig. S6 a breakdown of the step sizes leading to the intermediate configurations shown in Fig. S5. First, we note that the intermediate configuration in the CBDEA unfolding pathway concentrates at a step size around 7 nm, as shown in black in Fig. S6(A). The intermediate pathway corresponding to the DCEBA pathway is observed

---

*P.C. and G.Y. made equal contributions to this work
†Corresponding author: parkhs@bu.edu

to be concentrated around 12 nm as shown in green in Fig. S6(A), while the DCBEA intermediate step size has a broad distribution between about 8-12 nm, as shown in red in Fig. S6(B). Finally, the two state unfolding pathway shows a peak around 20 nm, in blue in Fig. S6(B). The combination of all four of these unfolding pathways is shown in the gold solid line in both Figs. S6(A) and (B), which illustrates that the most common intermediate configuration step sizes are concentrated around 7 and 12 nm lengths, similar to previous experimental studies on the mechanical unfolding of ubiquitin [5].

We show the disconnectivity tree structures of two representative unfolding pathways leading to two-state unfolding in Fig. S7, where the leaves in the disconnectivity structures represent local minima that were found during the unfolding process, where the branches connecting each set of leaves represents the minimum energy barrier that must be crossed between those local minima. Both tree structures show similarities, including the energy of the unfolded configuration.

---

[1] Cao, P., Li, M., Heugle, R. J., Park, H. S., and Lin, X. (2012). A self-learning metabasin escape algorithm and the metabasin correlation length of supercooled liquids. *Phys. Rev. E.*, 86:016710.

[2] Cao, P., Park, H. S., and Lin, X. (2013). Strain-rate and temperature-driven transition in the shear transformation zone for two-dimensional amorphous solids. *Phys. Rev. E*, 88:042404.

[3] Irbäck, A., Mitternacht, S., and Mohanty, S. (2005). Dissecting the mechanical unfolding of ubiquitin. *Proc. Natl. Acad. Sci. U.S.A.*, 102(38):13427–13432.

[4] Kushima, A., Lin, X., Li, J., Eapen, J., Mauro, J. C., Qian, X., Diep, P., and Yip, S. (2009). Computing the viscosity of supercooled liquids. *J. Chem. Phys.*, 130:224504.

[5] Schlierf, M., Li, H., and Fernandez, J. M. (2004). The unfolding kinetics of ubiquitin captured with single-molecule force-clamp techniques. *Proc. Natl. Acad. Sci. U.S.A.*, 101(19):7299–7304.

[6] Stirnemann, G., Giganti, D., Fernandez, J. M., and Berne, B. J. (2013). Elasticity, structure, and relaxation of extended proteins under force. *Proc. Natl. Acad. Sci. U.S.A.*, 110(10):3847–3852.

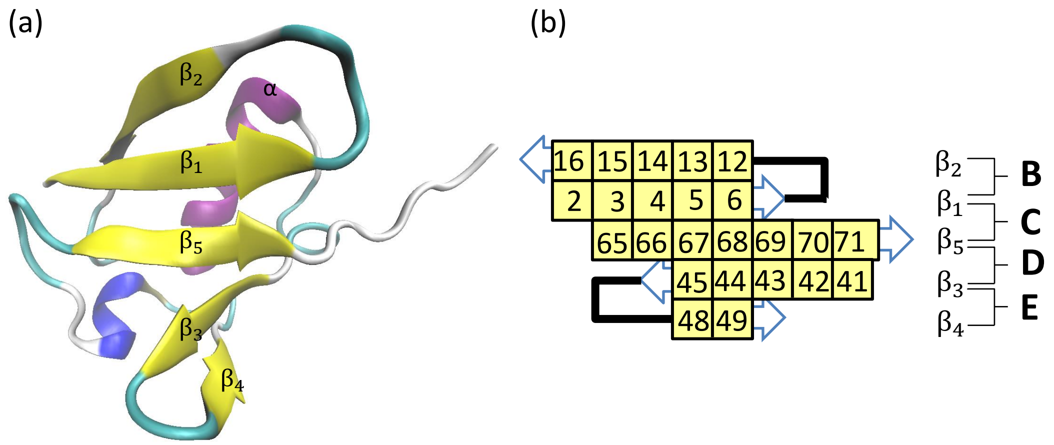

FIG. 1: (a) Schematic illustration of the native structure of ubiquitin (b) Structures of B,C, D and E consist of pairs of strands [3].

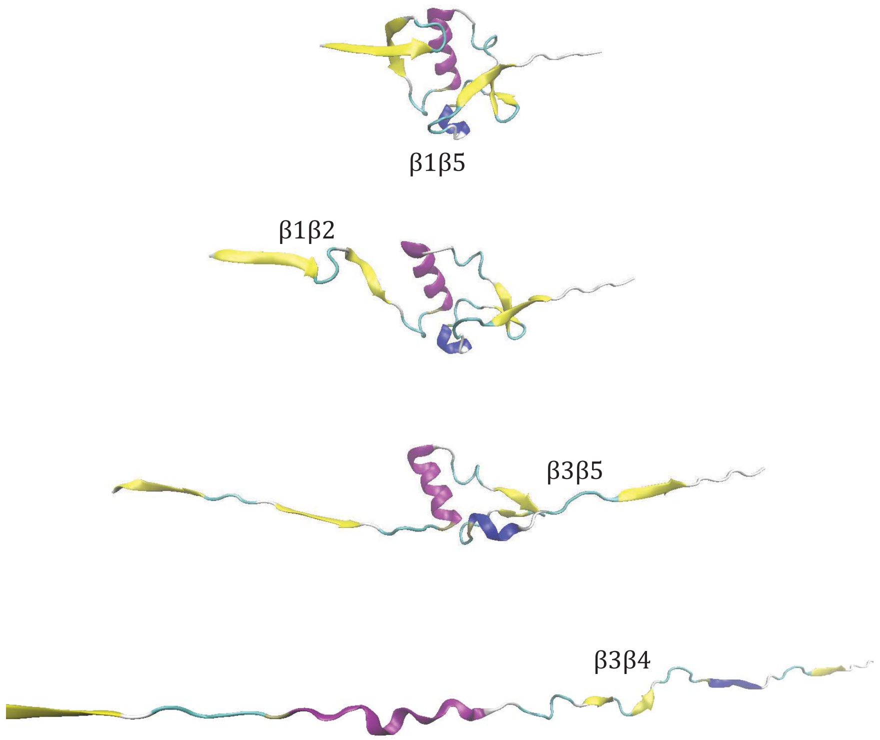

FIG. 2: Unfolding pathway at 350 pN for both SLME and SMD simulations.

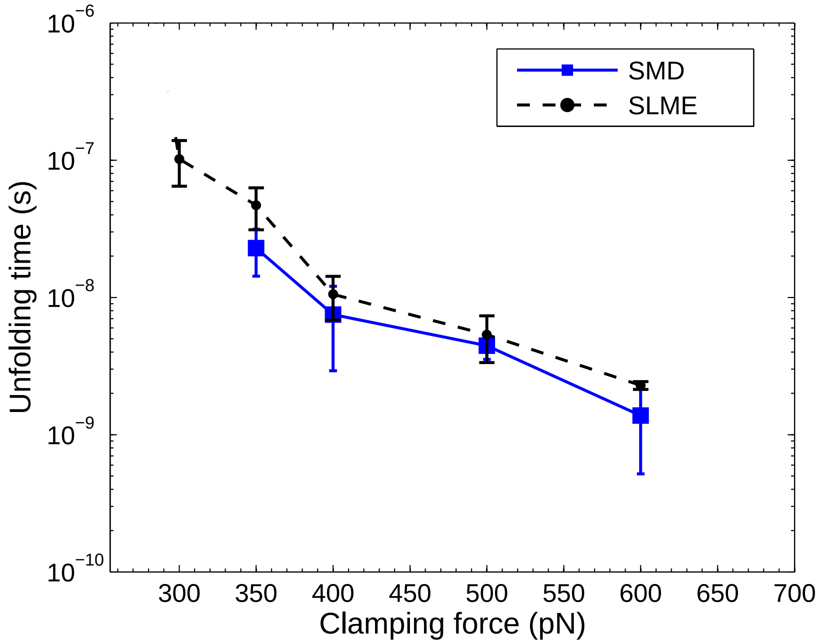

FIG. 3: Unfolding time for different clamping forces as computed using SMD, and SLME for ubiquitin.

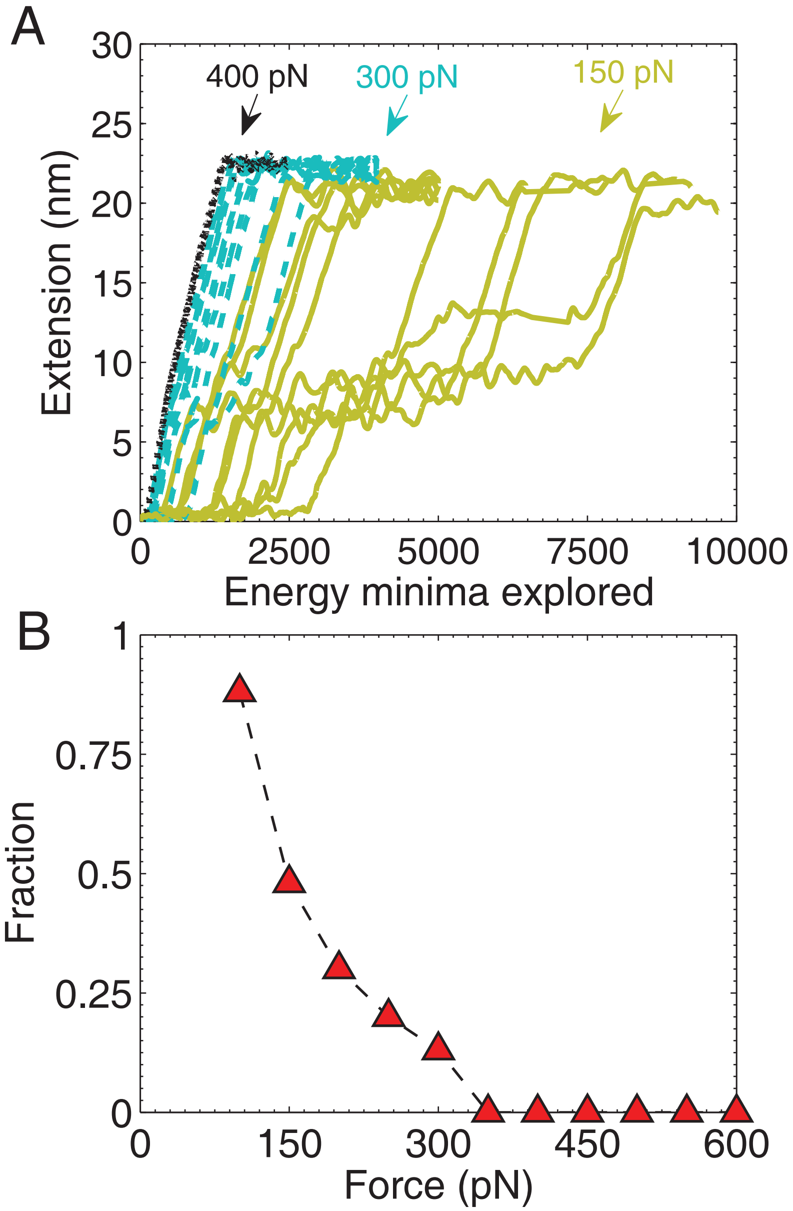

FIG. 4: (A) End-to-end extension as a function of the energy minima explored along the unfolding pathways for different clamping forces; (B) Fraction of SLME unfolding simulations that exhibit an intermediate state as a function of clamping force.

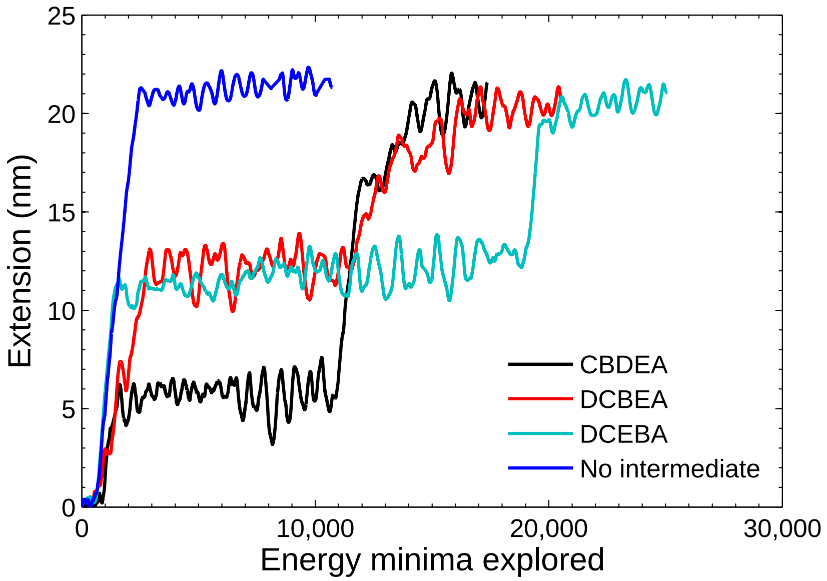

FIG. 5: Unfolding step size vs. local minima for four most common unfolding pathways. (CBDEA:$\beta_1\beta_5 \rightarrow \beta_1\beta_2 \rightarrow \beta_3\beta_5 \rightarrow \beta_3\beta_4 \rightarrow \alpha_1$, DCBEA: $\beta_3\beta_5 \rightarrow \beta_1\beta_5 \rightarrow \beta_1\beta_2 \rightarrow \beta_3\beta_4 \rightarrow \alpha_1$, DCEBA: $\beta_3\beta_5 \rightarrow \beta_1\beta_5 \rightarrow \beta_3\beta_4 \rightarrow \beta_1\beta_2 \rightarrow \alpha_1$)

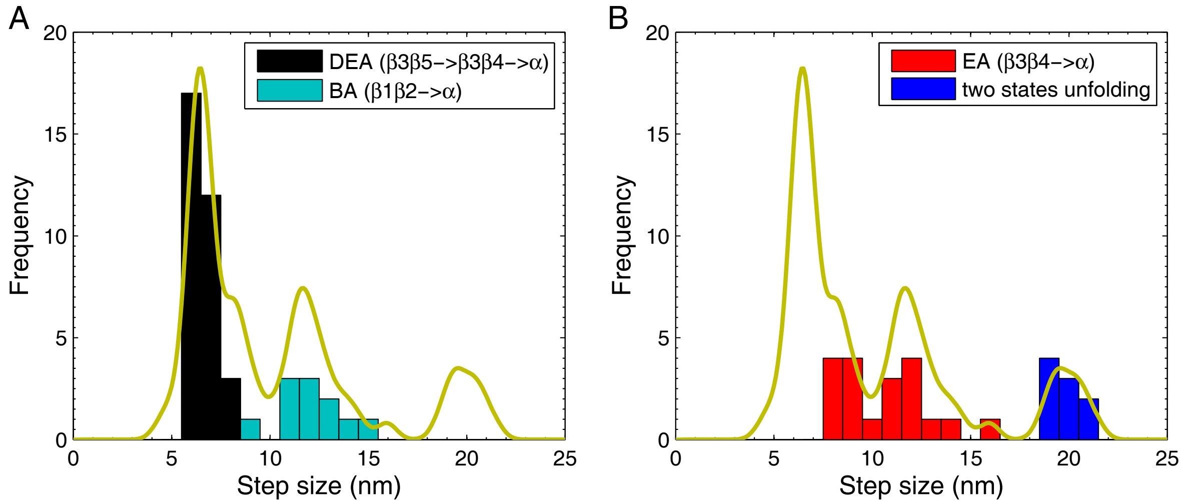

FIG. 6: Distribution of step sizes for four most common unfolding pathways at 100 pN clamping force as obtained using SLME.

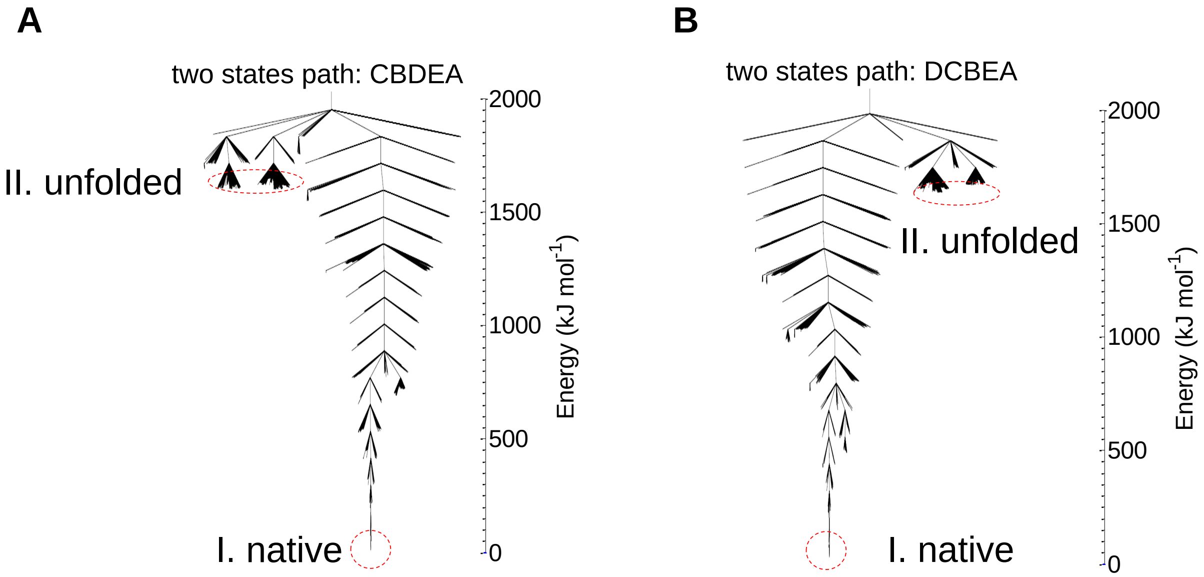

FIG. 7: Disconnectivity graph of unfolding sequence of (A) CBDEA and (B) DCBEA, both for two-state unfolding at 100 pN clamping force as obtained using SLME.
